# Correlation Between the NLRP3 Inflammasome and the Prognosis of Patients With LSCC

**DOI:** 10.3389/fonc.2019.00588

**Published:** 2019-07-02

**Authors:** Yi Xue, Huai-Dong Du, Di Tang, Duo Zhang, Jian Zhou, Chang-Wen Zhai, Cun-Cun Yuan, Chi-Yao Hsueh, Sheng-Jie Li, Yu Heng, Lei Tao, Li-Ming Lu

**Affiliations:** ^1^Department of Otolaryngology, Eye Ear Nose and Throat Hospital, Fudan University, Shanghai, China; ^2^Department of Pathology, Eye, Ear, Nose and Throat Hospital, Fudan University, Shanghai, China; ^3^Department of Clinical Laboratory, Eye, Ear, Nose and Throat Hospital, Fudan University, Shanghai, China; ^4^Shanghai Institute of Immunology, Shanghai Jiao Tong University School of Medicine, Shanghai, China

**Keywords:** laryngeal squamous cell carcinoma, NLRP3, inflammasome, immunohistochemistry, prognosis

## Abstract

**Background:** NLRP3 inflammasome is an inflammatory mediator. The expression of NLRP3 inflammasome is associated with the development of various tumors and is closely related to the prognosis of tumors. However, the role of NLRP3 inflammasome in laryngeal squamous cell carcinoma (LSCC) remains unclear. This study aim to investigate the influence of NLPR3 inflammasome expression in LSCC, and especially the NLRP3 inflammasome expression level and the prognosis of LSCC after surgery in a Chinese population.

**Methods:** We used quantitative real-time PCR and immunohistochemical (IHC) staining to calculate the mRNA (20 patients, fresh tissue) and protein expression (104 patients, paraffin tissue microarray) levels of the NLRP3 inflammasome (NLRP3/IL-18/IL-1β/ASC/caspase-1), respectively. We also analyzed the relationship between NLRP3 inflammasome expression levels and LSCC cancer tissues compared with adjacent normal tissues and the clinical features of LSCC. Kaplan–Meier survival curves of overall survival (OS) and disease-free survival (DFS) in LSCC patients were compared and analyzed under different expression levels of the NLRP3 inflammasome.

**Results:** Our results indicated that the mRNA expression of the NLRP3 inflammasome was higher in LSCC cancer tissues compared with adjacent normal tissues (*p* < 0.001). The IHC staining score also demonstrated that the expression of the NLRP3 inflammasome was higher than in the adjacent normal tissues (*p* < 0.001). The NLRP3 inflammasome expression also exhibited a close relationship with the clinicopathological characteristics (especially the stage of LSCC) of LSCC. Univariate Cox regression analysis and multivariate Cox regression analysis revealed that both NLRP3 and IL-1β had an increased risk of LSCC progression (*p* < 0.05). The Kaplan–Meier log rank test (OS and DFS) demonstrated that high expression of NLRP3/IL-18/IL-1β/ASC was statistically different than the low expression group (*p* < 0.05) of LSCC patients after surgery.

**Conclusion:** The high expression group of the NLRP3 inflammasome (NLRP3/IL-18/IL-1β/ASC) had a poorer prognosis (OS and DFS) than the low expression group of LSCC patients 5 years after surgery. The NLRP3 inflammasome (NLRP3/IL-18/IL-1β/ASC) may be used as an auxiliary indicator to predict LSCC patient prognosis after surgery.

## Introduction

Laryngeal squamous cell carcinoma (LSCC) is the most common pathological type (>95%) of laryngeal cancer (1). It is also the type of head and neck cancer that has the highest rate of mortality and morbidity (2). The most important factors associated with the development of laryngeal cancer may be tobacco smoking, alcohol consumption, old age, and male gender (3). Most patients are men. Typically, the ratio for men is 4:1, and the ratio for women is 20:1 (4). The application of more therapeutic methods and treatment concepts has greatly improved in the past decade, and the incidence of LSCC has decreased. However, the 5-year overall survival (OS) rate dropped from 66 to 63% (5). The cellular and molecular mechanisms of LSCC remain unclear. While, Inflammasome-mediated cancer recently attracted great interest, and NLRP3 inflammasome plays an important role in the development of a variety of solid tumors, including head and neck squamous cell carcinoma (HNSCC) (6) and oral squamous cell carcinoma (OSCC) (7). Inflammasomes play an important role in carcinogenesis and tumor progression (8). However, whether the NLRP3 inflammasome is involved in LSCC progression still unclear. In this study, we investigated the relationship between the prognosis of LSCC and NLRP3 inflammasome expression.

The NOD-like receptor family pyrin domain–containing 3 (NLRP3) inflammasome consists of an NLRP3 scaffold and a caspase-1 and caspase-recruitment domain (ASC) adaptor. It is the most widely studied NLR family member (9). NLRP3, which can be activated by multiple activators, including LPS, ATP, mitochondrial ROS (10). Activated NLRP3 recruits ASC and pro-caspase-1 to participate in the formation of NLRP3 inflammasome, while NLRP3 inflammasome can activate pro-Caspase-1. Activated caspase-1 can cleave pro-IL-1β, and pro-IL-18, allowing them to mature (IL-1β and IL-18) to involved in innate immunity (11). The NLRP3 inflammasome components contains several proteins, including NLRP3, caspase-1, ASC, IL-18, and IL-1β. NLRP3 inflammasome may have a dual role in tumors (12). Recent studies suggest that most experimental and clinical studies support NLRP3 inflammasome to promote tumor development. Blockade of NLRP3 may be a promising immunotherapy for pancreatic carcinoma, which drives macrophage-induced adaptive immune suppression (13). Previous studies also demonstrated that NLRP3 inflammasome, and its complex (IL-11β and IL-18) promote tumor growth, proliferation, invasion and metastasis in lung cancer, melanoma, breast cancer and HNSCC (12). However, studies on the association between the NLRP3 inflammasome and LSCC without distant metastasis are rare.

Thus we study the NLRP3 inflammasome expression in LSCC. And this study provided evidence that high NLRP3 inflammasome (NLRP3/IL-18/IL-1β/ASC) expression in LSCC cancer tissues suggests a poor prognosis of LSCC without distant metastasis in a Chinese population, which may help identify a new prognosis factor for LSCC.

## Materials and Methods

### Ethical Approval

The study was performed according to the principles of the Declaration of Helsinki, and the Medical Research Council of the Eye, Ear, Nose and Throat Hospital, Fudan University, Shanghai, China approved this study (No. KJ2008-01). Written informed consent was obtained and approved for all patients.

### Patient Information and Tissue Microarray (TMA) Construction

**Figure 1:**
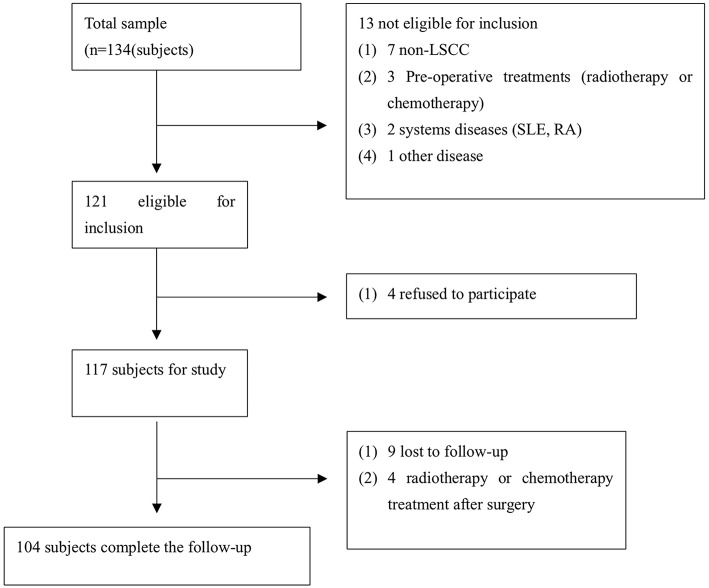
The study cohort flow diagram.

TMA were serially sliced (Wuhan biossci biotechnology Co.; Ltd, Wuhan, China) and consisted of LSCC tissue specimens containing cancer tissue and adjacent normal tissues that were collected between September 2013 and January 2015 in our hospital. Each of the TMA sections consisted of 4-μm-thick tissues. The inclusion criteria were established for the enrolled cases (Figure 1). A total of 104 cases were eligible for enrollment in this study (Figure 1), including all cases without radiotherapy or chemotherapy treatment (before and after surgery). The tissue used for RNA extraction was obtained from 20 pairs of LSCC patients, and samples were collected at the Eye, Ear, Nose, and Throat Hospital, Fudan University, Shanghai, China from January 2018 to July 2018 (Table 1). The clinicopathological findings of the specimens obtained from the patients in the cancer group revealed that all of the pathological cell types were squamous cell carcinoma. The histological grade of the LSCC cases was categorized according to the degree of differentiation. The Eighth Edition of the American Joint Committee on Cancer (AJCC) Tumor, Node, Metastasis (TNM) guidelines were used to stage the laryngeal tumors. The following definitions were also used to categorize smokers and non-smokers and alcohol consumers and non-alcohol consumers: non-smokers and smokers (≥20 cigarettes/day for ≥1 year); and non-alcohol consumers and alcohol consumers (≥200 ml/day for ≥1 year).

**Table 1:** Clinicopathological characteristics of patients with LSCC [TAM (104 patients), fresh tissues used for qPCR (20 patients)].

**Characteristics**	**TAM**	**RNA isolation**
	**Number (%)**	**Number (%)**
**Age (y)**		
<60 years	35 (33.7)	7 (35.0)
≥60years	69 (66.3)	13 (65.0)
**Gender**		
Female	1 (1.0)	1 (5.0)
Male	103 (99.0)	19 (95.0)
**Smoking**		
No	31 (29.8)	8 (40.0)
Yes	73 (70.2)	12 (60.0)
**Alcohol consumption**		
No	45 (43.3)	10 (50.0)
Yes	59 (56.7)	10 (50.0)
**Stage of LSCC**		
I	15 (14.4)	1 (5.0)
II	24 (23.1)	7 (35.0)
III	36 (34.6)	8 (40.0)
IV	29 (27.9)	4 (20.0)
**Typing of LSCC**		
Glottic type	50 (48.0)	8 (40.0)
Supraglottic type	45 (43.3)	10 (50.0)
Subglottic type	9 (8.7)	2 (10.0)
**Tumor diameter**		
<3cm	49 (47.1)	8 (40.0)
≥3cm	55 (52.9)	12 (60.0)
**Histological grade**		
SCC I	23 (22.1)	4 (20.0)
SCC II	68 (65.4)	13 (65.0)
SCC III	13 (12.5)	3(15.0)
**Lymph node**		
N0	66 (63.5)	11(55.0)
N1	21 (20.2)	3 (15.0)
N2	17 (16.3)	6 (30.0)
**Distant metastasis**		
M0	104 (100.0)	20 (100.0)
M1	0 (0.0)	0 (0.0)

### Total RNA Isolation and Reverse Transcription Quantitative Polymerase Chain Reaction (RT-qPCR)

We extracted the total RNA from fresh tissues of LSCC patients using TRIzol reagent (Invitrogen, Carlsbad, CA, USA). The extracted RNA had an absorbance range of 1.8–2.1 as measured using NanoDrop 2000 Spectrophotometer software. A volume of 2 μl RNA in a 10-μl reaction system was used to synthesize complementary DNA using a PrimeScript RT Reagent kit (DRR063A; Takara, Bio, Inc., Otsu, Japan). RNA-free water was used to dilute the obtained cDNA, which was diluted to the appropriate concentration and stored at −20° for experimental use. The same PrimeScript RT reagent kit was used for the RT-qPCR was used to detect NLRP3, IL-18, IL-1β, ASC, caspase-1, and glyceraldehyde 3-phosphate dehydrogenase (GAPDH). Applied Biosystems 7500 Fast Real-Time PCR System software version 2.0.6 (Thermo Fisher Scientific, Inc.) was used to analyze the data. GAPDH is a standardized reference gene that is used to evaluate the expression level of the target gene. The 2^−Δ*Cq*^ method was used to analyze the acquired data. All of the primers were designed based on search information that was obtained from the National Center for Biotechnology Information database. The following primers were used: GAPDH: forward 5′-GGTCGGAGTCAACGGATTTGGTCG-3′, reverse 5′-CCTCCGACGCCTGCTTCACCAC-3′; NLRP3: forward 5′-CCATCGGCAAGACCAAGA-3′, reverse 5′-ACAGGCTCAGAATGCTCATC-3′; IL-18: forward 5′-TCTTCATTGACCAAGGAAATCGG-3′, reverse 5′-TCCGGGGTGCATTATCTCTAC-3′; IL-1β, forward 5′-TACGAATCTCCGACCACC ACTACAG-3′, reverse 5′-TGGAGGTGGAGAGCTTTCAGTTCATATG-3′; and ASC: forward 5′-TGGATGCTCTGTACGGGAAG-3′, reverse 5′-CCAGGCTGGTGTGAAACTGAA-3′. Three replicates were included for each experiment, and the experiments were validated at least three times.

### Immunohistochemical (IHC) Staining and Evaluation of IHC Staining

The IHC staining process was basically performed under the same conditions for all specimens, with the exception of the use of different primary antibodies. The primary antibodies included anti-NLRP3 antibody (Abcam, no. ab214185) diluted 1:200, anti-IL-18 antibody (Abcam, no. ab68435) diluted 1:200, anti-IL-1β antibody (Abcam, no. ab2105) diluted 1:100, ASC (ProteinTECH, no. 10500-1-AP) diluted 1:300, and anti-caspase-1 antibody (Abcam, no. ab1872) diluted 1:50 at 4°C overnight. TMA was rinsed three times using phosphate-buffered saline (PBS). Tissues in the TMA were incubated with a secondary antibody (undiluted HRP rabbit/mouse; Dako, REAL^TM^ EnVision^TM^ detection system) for 30 min at room temperature. The stained area was washed three times with PBS, and the subsequent staining step was performed. The following criteria and methods were used for IHC scoring. Image-Pro Plus version 6.0 software (Media Cybernetics, Inc., Bethesda, MD, USA) was used to evaluate the intensity score (IS) of IHC by calculating the integrated optical density (IOD) three times in each field, and the IOD/the total area of each field was also calculated simultaneously. Specific IS information for IOD distribution in the cancer and adjacent normal tissues are shown in **Figures 3A–E**. According to the IOD value, the staining IS values were 0 (–), 1 (+), 2 (++), and 3 (+++). We divided the staining proportion score (PS) into four levels: 0 (0%); 1 (1–25%); 2 (26–50%); 3 (51–75%); and 4 (76–100%). The IS multiplied by PS was the final result of the staining score (14). The expression of the NLRP3 inflammasome (NLRP3, IL-18, IL-1β, ASC, and caspase-1) was categorized into a low expression group (0–6) and a high expression group (7–12) in this study according to the staining score results.

### Statistical Analysis

The chi-square test and Kaplan–Meier log rank test in IBM SPSS Statistics version 23.0 software (SPSS Inc., New York, NY, USA) were used to analyze the data obtained from the study, especially the differences in the NLRP3 expression levels between the cancer tissues and the adjacent normal tissues and the 5-year OS and disease free survival (DFS) rate of LSCC patients after surgery. GraphPad Prism version 7.0 software (GraphPad Software, Inc., San Diego, CA, USA) was used to analyze the differences in RNA expression levels between the cancer tissues and adjacent normal tissues. *P* < 0.05 indicated a statistically significant difference.

## Results

### The Expression Level of the NLRP3 Inflammasome Was Higher in the LSCC Tumor Tissues Than the Adjacent Normal Tissues

**Figure 2:**
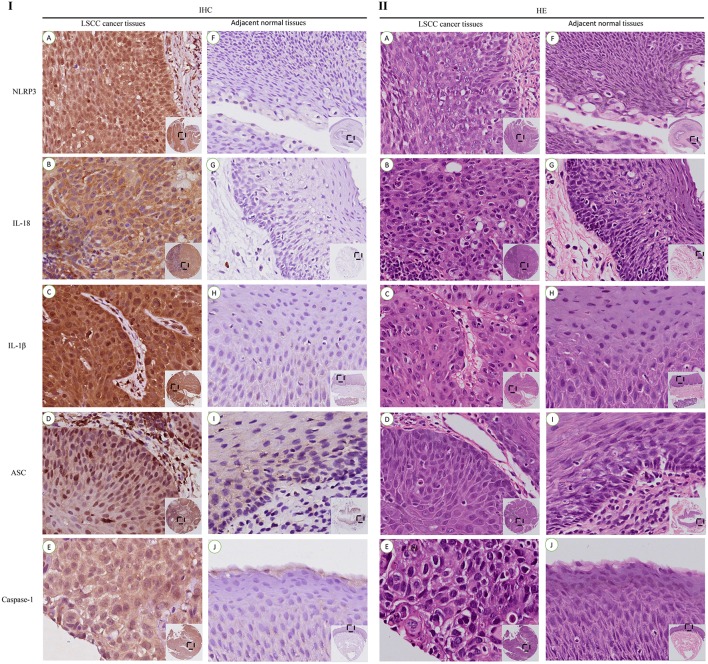
IHC staining and corresponding hematoxylin and eosin (HE) staining of NLRP3 inflammasome expression in LSCC tissues and adjacent normal tissues (200x).IHC staining of the NLRP3 inflammasome in LSCC tissues **(I)**: **(A)** NLRP3; **(B)** IL-18; **(C)** IL-1β; **(D)** ASC; **(E)** Caspase-1. Staining of the NLRP3 inflammasome in the adjacent normal tissues **(I)**: **(F)** NLRP3; **(G)** IL-18; **(H)** IL-1β; **(I)** ASC; **(J)** Caspase-1. HE Staining of the NLRP3 inflammasome in the LSCC tissues **(II)**: **(A)** NLRP3; **(B)** IL-18; **(C)** IL-1β; **(D)** ASC; **(E)** Caspase-1. HE Staining of the NLRP3 inflammasome in the adjacent normal tissues **(II)**: **(F)** NLRP3; **(G)** IL-18; **(H)** IL-1β; **(I)** ASC; **(J)** Caspase-1.

**Figure 3:**
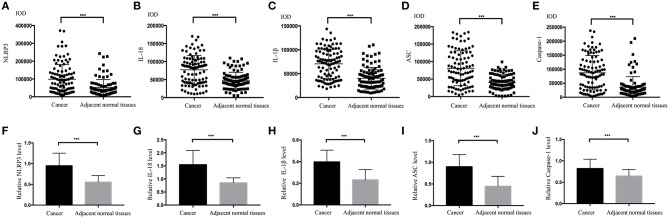
Comparisons of the IOD values for the NLRP3 inflammasome. **(A)** NLRP3; **(B)** IL-18; **(C)** ASC; **(D)** IL-1β; **(E)** Caspase-1 for the LSCC tissues and the adjacent normal tissues. Comparisons of the mRNA expression levels of the NLRP3 inflammasome for the LSCC tissues and the adjacent normal tissues using RT-qPCR **(F)**NLRP3; **(G)**IL-18; **(H)**ASC; **(I)** IL-1β; **(J)** Caspase-1. (all results, *P < 0.05, **P < 0.01, ***P < 0.001).

The IHC staining of 104 cases of LSCC tissues and the corresponding adjacent normal tissues revealed positive staining for the NLRP3 inflammasome components (NLRP3, IL-18, IL-1β, ASC, and caspase-1) primarily in the nucleus and cytoplasm of LSCC cells (Figures 2A–E). Each photograph of IHC also had its corresponding hematoxylin and eosin (H&E) staining picture. This study used negative control group for each staining group, but no positive staining was observed for the control group (data not shown), which ensured the accuracy of the IHC staining results. The IOD results between the two groups [cancer tissue group (Figures 2A–E) and adjacent normal tissue group (Figures 2F–J)] were significantly different (Figures 3A–E, *P* < 0.001). The chi-square test was used to determine significant differences between the two groups, and a statistically significant difference was found between the two groups (Table 2, *P* < 0.001). NLRP3 inflammasome expression was validated using RT-qPCR experiments to study the mRNA levels of the NLRP3 inflammasome in cancer tissues and the corresponding adjacent normal tissues from another cohort of 20 LSCC patients (Table 1). The results showed that the mRNA expression of the NLRP3 inflammasome markedly increased in the cancer tissues compared to the adjacent normal tissues (Figures 3F–J*, P* < 0.001). This result indicates that the expression of NLRP3 inflammasome was higher in the cancer tissue group than the adjacent normal tissue group at the gene and protein levels.

**Table 2:** Expression of the NLRP3 inflammasome in the LSCC tissues and adjacent normal tissues.

**Feature**	**Cancerous tissues**		**Adjacent normal tissues**		**χ^2^**	***P***
	***N***	***%***	***n***	***%***		
**NLRP3**						
High	49	47.1	8	7.7		
Low	55	52.9	96	92.3	40.624	0.000
**IL-18**						
High	57	55.9	14	13.7		
Low	45	44.1	88	86.3	39.945	0.000
**IL-1β**						
High	55	55.6	15	15.2		
Low	44	44.4	84	84.8	35.357	0.000
**ASC**						
High	55	59.8	10	10.9		
Low	37	40.2.	82	89.1	48.171	0.000
**Caspase-1**						
High	51	52.6	9	9.3		
Low	46	47.4	88	90.7	42.564	0.000

*P < 0.001 were analyzed by Pearson's χ^2^ test*.

### The Significance of the Expression Levels of NLRP3 Inflammasome Components With One Another in Cancer Tissues

**Figure 4:**
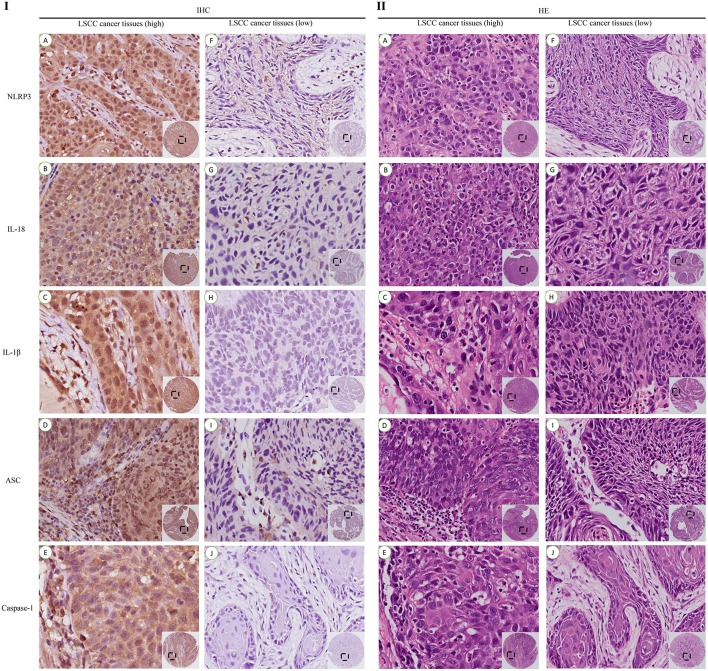
IHC and HE staining of the high and low expression levels of the NLRP3 inflammasome in LSCC tissues (200x). IHC staining of the high expression group **(I)**: **(A)** NLRP3; **(B)** IL-18; **(C)** IL-1β; **(D)** ASC; **(E)** Caspase-1. IHC staining of the low expression group **(I)**: **(F)** NLRP3; **(G)** IL-18; **(H)** IL-1β; **(I)** ASC; **(J)** Caspase-1. HE staining of the high expression group **(II)**: **(A)** NLRP3; **(B)** IL-18; **(C)** IL-1β; **(D)** ASC; **(E)** Caspase-1. HE staining of the low expression group **(II)**: **(F)** NLRP3; **(G)** IL-18; **(H)** IL-1β; **(I)** ASC; **(J)** Caspase-1.

LSCC cancer tissues were divided into a high expression group (Figures 4A–E) and a low expression group (Figures 4F–J) based on the previous IHC staining score, and the chi-square test was used to calculate the *P*-value. The relationships between factors in the high expression of NLRP3 inflammasome (NLRP3, IL-18, IL-1β, ASC, and caspase-1) were analyzed (Table 3). All of the results revealed a significant *P*-value (*P* < 0.05), except the relationship between ASC and IL-1β (*P*>0.05). However, the relationships between the NLRP3 protein and other inflammasome components (IL-18, IL-1β, ASC, and caspase-1) were more significantly different (*P* < 0.001).

**Table 3:** Correlations among the NLRP3 inflammasome components in LSCC tissues.

**NLRP3**	**IL-18**				**IL-1β**				**ASC**				**Caspase-1**						**ASC**			
	**High**	**Low**	**χ^2^**	***P***	**High**	**Low**	**χ^2^**	***P***	**High**		**Low**	**χ^2^**	***P***	**High**	**Low**	**χ^2^**	***P***	**IL-1β**	**High**	**Low**	**χ^2^**	***P***
High	43	6			39	10			41	8			42	7			High	38	20			
low	14	41	40.607	0.000	19	36	21.317	0.000	20	35	23.916	0.000	14	41	37.863	0.000	low	23	23	2.547	0.110	
**IL-18**	**IL-1β**				**ASC**				**Caspase-1**				**Caspase-1**					**Caspase-1**				
	**High**	**Low**	**χ^2^**	***P***	**High**	**Low**	**χ^2^**	***P***	**High**	**Low**	**χ^2^**	***P***	**ASC**	**High**	**Low**	**χ^2^**	***P***	**IL-1β**	**High**	**Low**	**χ^2^**	***P***
High	40	17			44	13			41	16			High	43	18			High	43	15		
low	18	27	9.335	0.002	17	28	16.252	0.000	12	32	19.857	0.000	low	13	30	16.449	0.000	low	13	33	21.726	0.000

*P -value were analyzed by Pearson's χ^2^ test*.

### The Relationship Between NLRP3 Inflammasome Expression and the Clinicopathological Characteristics of LSCC

To study the potential relationship between NLRP3 inflammasome expression and LSCC, the clinicopathological features of LSCC were analyzed (Table 4). Several risk factors associated with the onset of LSCC (smoking and alcohol consumption) were selected. The clinical stage of LSCC, the histological grade of LSCC, the maximum diameter of the cancer tumor and typing of LSCC were also assessed. The results indicated that the NLRP3 inflammasome expression levels with alcohol consumption (IL-18, χ^2^ = 5.073, *P* = 0.024), the stage of LSCC (NLRP3, χ^2^ = 9.526, *P* = 0.022; IL-18, χ^2^ = 12.089, *P* = 0.007, IL-1β, χ^2^ = 10.190, *P* = 0.017; ASC, χ^2^ = 23.659, *P* < 0.001; caspase-1, χ^2^ = 7.916, *P* = 0.049), histological grade (NLRP3, χ^2^ = 7.708, *P* = 0.029; IL-18, χ^2^ = 7.267, *P* = 0.023; IL-1β, χ^2^ = 13.959, *P* = 0.001), and tumor diameter (NLRP3, χ^2^ = 4.007, *P* = 0.045; IL-18, χ^2^ = 5.342, *P* = 0.021; ASC, χ^2^ = 5.244, *P* = 0.022) exhibited significant differences. More detailed information is shown in Table 4. These results suggest that the NLRP3 inflammasome, which may affect the development of LSCC, is related with the clinicopathological characteristics of LSCC.

**Table 4:** Correlations between the NLRP3 inflammasome expression levels in the LSCC tissues and the clinicopathologic features of LSCC.

**Feature**	**Age(year)**				**Smoking**				**Alcohol consumption**				**Stage of LSCC**						**Histological grade**					**Tumor diameter**				**Typing of LSCC**				
	**≥60**	** <60**	**χ^2^**	***P***	**NO**	**Yes**	**χ^2^**	***P***	**NO**	**Yes**	**χ^2^**	***P***	**I**	**II**	**III**	**IV**	**χ^2^**	***P***	**SCC I**	**SCC II**	**SCC III**	**χ^2^**	***P***	** <3cm**	**≥3cm**	**χ^2^**	***P***	**Glottic type**	**Supraglottic type**	**Subglottic type**	**χ^2^**	***P***
**NLRP3**																																
High	32	17			12	37			17	32			3	8	21	17			5	36	8			18	31	4.007	0.045	24	19	6		
low	37	18	0.045	0.832	19	36	1.252	0.263	28	27	2.776	0.096	12	16	15	12	9.526	0.022	18	32	5	7.708	0.029	31	24			26	26	3	1.794	0.428
**IL-18**																																
High	38	19			13	44			19	38			5	8	23	21			7	41	9			21	36			28	24	5		
low	31	16	0.006	0.939	18	29	2.954	0.086	26	21	5.073	0.024	10	16	13	8	12.089	0.007	16	27	4	7.267	0.023	28	19	5.342	0.021	22	21	4	0.140	0.956
**IL-1β**																																
High	36	22			13	45			25	33			6	8	24	20			5	45	8			27	31			28	24	6		
low	33	13	1.074	0.300	18	28	3.426	0.064	20	26	0.001	0.969	9	16	12	9	10.190	0.017	18	23	5	13.959	0.001	22	24	0.614	0.433	22	21	3	0.538	0.837
**ASC**																																
High	42	19			17	44			25	36			4	13	25	19			10	42	9			23	38			29	25	7		
low	27	16	0.415	0.519	14	29	0.115	0.735	20	23	0.314	0.575	11	11	11	10	23.659	0.000	13	26	4	2.965	0.230	26	17	5.244	0.022	21	20	2	1.441	0.505
**Caspase1**																																
High	37	19			15	41			23	33			4	11	21	20			9	40	7			24	32			27	22	7		
low	32	16	0.004	0.949	16	32	0.530	0.467	22	26	0.239	0.625	11	13	15	9	7.916	0.049	14	28	6	2.682	0.262	19	23	0.055	0.814	23	23	2	2.415	0.300

*P-value were analyzed by Pearson's χ^2^ test*.

### The Higher NLRP3 Inflammasome (NLRP3/IL-18/IL-1β/ASC) Expression in Cancer Tissues Was Associated With Poor Survival of LSCC Patients

LSCC cancer tissues were divided into two groups based on the IHC scores. One group exhibited high expression of the NLRP3 inflammasome in LSCC cancer tissues, and the other group exhibited low expression of the NLRP3 inflammasome in LSCC cancer tissues (Figure 4). Univariate and multivariate Cox regression analyses (including age, smoking, alcohol consumption, stage of LSCC, histological grade, and tumor diameter) were also used to determine the relationship between NLRP3 inflammasome expression and the survival of LSCC patients. Univariate Cox regression analysis showed that LSCC patients with stage of LSCC (hazard ratio (HR) = 1.517, 95% confidence interval (CI) 1.091–2.111, *P* = 0.013), histological grade (HR = 2.119, 95%CI 1.241–3.620, *P* = 0.006), tumor diameter (HR = 2.686, 95%CI 1.385–5.211 *P* = 0.003), NLRP3 (HR = 2.196, 95%CI 1.183–4.076 *P* = 0.013), IL-18 (HR = 2.066, 95%CI 1.085–3.932 *P* = 0.027), IL-1β (HR = 2.041, 95%CI 1.073–3.882 *P* = 0.030), and ASC (HR = 1.946, 95%CI 1.010–3.748, *P* = 0.047) had an increased risk of LSCC progression (Table 5). Multivariate Cox regression analysis showed that LSCC patients with histological grade (HR = 1.873, 95%CI 1.021–3.436, *P* = 0.043), tumor diameter (HR = 2.321, 95%CI 1.160–4.644, *P* = 0.017), NLRP3 (HR = 1.940, 95%CI 1.026–3.667, *P* = 0.041), and IL-1β (HR = 2.128, 95%CI 1.046–4.332, *P* = 0.037) also had an increased risk of LSCC progression (Table 5).

**Table 5:** Univariate/multivariate Cox proportional regression survival analysis.

	**Univariate analysis**			**Multivariate analysis**		
**Variable**	**HR**	**95% CI**	***P***	**HR**	**95% CI**	***P***
Age	0.935	0.497–1.759	0.836			
Smoking	1.030	0.527–2.014	0.931			
Alcohol consumption	1.093	0.593–2.014	0.776			
Typing of LSCC	1.117	0.697–1.790	0.646			
Stage of LSCC	1.517	1.091–2.111	0.013	1.353	0.945–1.939	0.099
Histological grade	2.119	1.241–3.620	0.006	1.873	1.021–3.436	0.043
Tumor diameter	2.686	1.385–5.211	0.003	2.321	1.160–4.644	0.017
**NLRP3**						
High vs. Low	2.196	1.183–4.076	0.013	1.940	1.026–3.667	0.041
**IL-18**						
High vs. Low	2.066	1.085–3.932	0.027	1.713	0.872–3.368	0.118
**IL-1β**						
High vs. Low	2.041	1.073–3.882	0.030	2.128	1.046–4.332	0.037
**ASC**						
High vs. Low	1.946	1.010–3.748	0.047	1.735	0.885–3.402	0.109
**Caspase-1**						
High vs. Low	1.598	0.857–2.982	0.141	1.425	0.756–2.685	0.273

**Figure 5:**
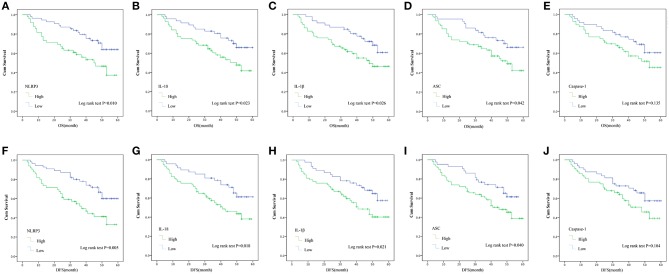
Kaplan–Meier with log rank test analyses of the influence of the expression of the NLRP3 inflammasome on the 5-year OS and DFS for 104 LSCC patients. OS: **(A)** NLRP3; **(B)** IL-18; **(C)** IL-1β; **(D)** ASC; **(E)**, Caspase-1. DFS: **(F)** NLRP3; **(G)** IL-18; **(H)** IL-1β; **(I)** ASC; **(J)** Caspase-1.

Kaplan-Meier with log rank test was used to evaluate the 5-year OS and DFS and the relationship between the level of NLRP3 inflammasome expression and the survival of LSCC patients for further research. The results indicate that LSCC patients with high NLRP3/IL-18/IL-1β/ASC expression had poorer OS compared to patients with low NLRP3/IL-18/IL-1β/ASC expression (Figure 5A, HR = 6.594, *P* = 0.010; Figure 5B, HR = 5.149, *P* = 0.023; Figure 5C, HR = 4.974, *P* = 0.026; Figure 5D, HR = 4.417, *P* = 0.042). The following 5-year survival rates after surgery in LSCC patients in different groups were calculated (according to OS). The high expression group vs. low expression group: NLRP3, 49.0 vs. 69.1%; IL-18, 50.9 vs. 70.2%; IL-1β, 51.7 vs. 69.6%; ASC, 52.5 vs. 69.8%; and caspase-1, 53.6 vs. 66.7%. The DFS results after 5 years in postoperative LSCC patients also demonstrated that LSCC patients with high NLRP3/IL-18/IL-1β/ASC expression had poorer OS compared to patients with low NLRP3/IL-18/IL-1β/ASC expression (Figure 5F, HR = 7.907, *P* = 0.005; Figure 5G, HR = 5.639, *P* = 0.018; Figure 5H, HR = 5.363, *P* = 0.021; Figure 5I, HR = 2.638, *P* = 0.040). However, no statistically significant difference in OS or DFS after surgery for LSCC patients was found between high caspase-1 expression and low caspase-1 expression (Figure 5E, HR = 2.234, *P* = 0.135; Figure 5J, HR = 2.638, *P* = 0.104). The following 5-year survival rates after surgery in LSCC patient in different groups were calculated (according to DFS). The high expression group vs. low expression group: NLRP3, 42.9 vs. 65.5%; IL-18, 45.6 vs. 66.0%; IL-1β, 46.6 vs. 65.2%; ASC, 47.5 vs. 65.1%; and caspase-1, 48.2 vs. 62.5%. The expression levels of caspase-1 in the OS and DFS results were not significantly different (OS, HR = 2.234, *P* = 0.135; DFS, HR = 2.638, *P* = 0.104). These results show that higher NLRP3 inflammasome (NLRP3/IL-18/IL-1β/ASC) expression in cancer tissues results in poor OS in LSCC patients compared to lower NLRP3 inflammasome (NLRP3/IL-18/IL-1β/ASC) expression.

## Discussion

The NLRP3 inflammasome was extensively studied in different tumors, and its role in malignant disease has become clearer. However, the present study is the first study to examine the expression of the NLRP3 inflammasome in LSCC tumor tissues and investigate the correlation between its expression and the prognosis of LSCC without distant metastasis in a Chinese population.

Chronic inflammation increases cancer risk, which impacts every step of tumorigenesis. Cancer-related inflammation is one of the seven hallmarks of cancer (15). The NLRP3 inflammasome is involved the progression of asthma (16) and inflammatory bowel diseases (17). The NLRP3 inflammasome also promotes tumor growth and metastasis in various cancers, such as OSCC (7), HNSCC (6), pancreatic carcinoma (13), and breast cancer (18). The NLRP3 inflammasome is a factor for epithelial-mesenchymal transition (EMT) in colon cancer cells (19). The findings of the present study suggest that NLRP3 inflammasome overexpression exerts a greater impact on LSCC tumor tissues than the corresponding adjacent normal tissues (Figures 3A–E), and mRNA expression of the NLRP3 inflammasome is higher in the tumor tissues than the adjacent normal tissues under the same conditions (Figures 3F–J). The findings reported in previous studies also support the findings in the present paper; NLRP3 inflammasome expression is higher in cancer tissues than adjacent normal tissues in other types cancers (6, 14, 20). Previous studies suggested that nuclear factor-kB (NF-kB) is activated by Toll-like receptor (TLR) signaling and upregulates the expression of NLRP3 and pro-IL-1β (21). Therefore, increased NLRP3 inflammasome expression may be associated with activation of NF-kB. These results suggest that the NLRP3 inflammasome plays an important role in the tumor microenvironment of LSCC.

To further investigate the role of the NLRP3 inflammasome components, the present study also analyzed the significance of the interrelationship between NLRP3 inflammasome components. There was no significant difference between ASC and caspase-1 (Table 3. *P* > 0.05). However, this result may be related to the sample size and will be further verified in future experiments. Almost all the results herein exhibited a significant difference (*P* < 0.05). The relationship of NLRP3 with IL-18, IL-1β, ASC and caspase-1 exhibited especially significant differences (*p* < 0.001). Our results are similar to Wei et al. (22) who indicated that the NLRP3 protein was closely related with inflammasome components. However, our results suggest that NLRP3 has a close relationship with other components (IL-18, IL-1β, ASC, and caspase-1) in LSCC. These findings also demonstrated a correlation between NLRP3 inflammasome expression level and the clinical parameters of LSCC (Table 4), especially with the stage of LSCC and histological grade and the NLRP3 protein with clinical parameters. The findings of the present study also suggest a statistically significant correlation between IL-18 and alcohol consumption (*P* < 0.05). This result may be because drinking alcohol increases inflammasome expression and promotes tumor development. Although this study did not find any statistically significant differences between smoking and NLRP3 inflammasome expression, the existence of a relationship cannot be excluded. Smoking may be a factor in the increased expression of inflammasomes in people who drink alcohol. Analysis of the relationships between NLRP3 inflammasome and clinical features revealed that NLRP3 exhibited a close relationship with the stage of LSCC. Therefore, we speculate that NLRP3 is involved in tumor aggressiveness. Some previous studies suggested that NLRP3 downregulation inhibited EMT and metastasis in *in vitro* and *in vivo* experiments, and the targeting of NLRP3 may be a promising strategy for the treatment of lung adenocarcinoma (23). Wang et al. (24) suggested that NLRP3 enhanced the proliferation and migration of A549 lung cancer cells via the release of IL-1β and IL-18 in an autocrine or paracrine manner. These studies may explain why NLRP3 promotes tumorigenesis and provide new ideas for studying the relationship between NLRP3 inflammasome expression and LSCC after surgery.

The present study used univariate and multivariate Cox regression (Table 5) to examine the relationship between NLRP3 inflammasome components and the OS and DFS of LSCC patients (Figure 5). The results showed that NLRP3 inflammasome components, dominated by NLRP3, may be a risk factor for the progression of LSCC. The results indicate a statistically significant correlation between the 5-year OS and DFS after surgery and the expression level of NLRP3 inflammasome (NLRP3/IL-18/ IL-1β/ASC) and the prognosis of LSCC patients (Figure 5). In short, the OS and DFS results show that high NLRP3 inflammasome expression (NLRP3/IL-18/ IL-1β/ASC) in LSCC cancer tissues forecasts a poor outcome compared to low NLRP3 inflammasome expression (NLRP3/IL-18/IL-1β/ASC) in cancer tissues (Figure 5, *P* < 0.05). The reason for the poor prognosis of LSCC patients with high NLRP3 inflammasome expression is not clear, and further research is needed. However, previous studies suggested that inflammasomes are one of the seven hallmarks of cancer (15), and its activation may lead to suppression of the anti-tumor immunity of NK and T cells (25). NLRP3 inflammasome expression may be associated with the occurrence of EMT (19), cancer stem cells self-renewal activation (6) and the overexpression of IL-18, which is related to increases in myeloid-derived suppressor cells (MDSCs) (25). These mechanisms may play important roles in the involvement of NLRP3 inflammasome expression in tumorigenesis. The findings reported in some previous studies also support these results. Benjamin et al. (26) indicated that the NLRP3 inflammasome played an unappreciated role via downstream lymphatics and S1PR1 signaling in macrophages, which promotes lymphangiogenesis and metastasis. Feng et al. (14) suggested that 5-FU-based adjuvant chemotherapy for OSCC was improved by the targeting of the ROS/NLRP3/IL-1β signaling pathway, and high NLRP3 expression was associated with poor clinical outcome in 5-FU-treated OSCC patients. These studies provide strong evidence that the NLRP3 inflammasome promotes tumorigenesis.

However, studies investigating different types of tumors report a different view of the role of NLRP3 in tumors. Zaki et al. (27) demonstrated that the NLRP3 inflammasome prevented the development of inflammation-associated colorectal tumorigenesis. Wei et al. (22) demonstrated that hepatocellular carcinoma (HCC) progression was associated with the deregulated expression of NLRP3 inflammasome components (including ASC, caspase-1, and IL-1β), and the NLRP3 inflammasome played a protective role in HCC. The reason for these different results may be related to the type of tumor and the role of the NLRP3 inflammasome in different tumor microenvironments. Additional work is needed to investigate the role of the NLRP3 inflammasome in different types of tumors.

There are some limitations in the present study. The sample size of clinical cases included was not sufficiently large. The incidence of laryngeal cancer in female patients is low, and the number of female cases included was relatively small. The follow-up time not sufficiently long. Therefore, there may be some bias in the results, and further research on the pathogenesis of NLRP3 inflammasome expression and LSCC is needed.

In summary, the present study found that NLRP3 inflammasome expression was higher in cancer tissues from LSCC patients without distant metastasis than adjacent normal tissues. The study also found a correlation between NLRP3 inflammasome expression and part of the clinicopathological characteristics of LSCC. High NLRP3 inflammasome expression level (NLRP3/IL-18/ IL-1β/ASC) suggested a poor prognosis for 5-year OS and DFS. The NLRP3 inflammasome (NLRP3/IL-18/ IL-1β/ASC) may be used as an auxiliary indicator to predict LSCC patient prognosis after surgery.

## Data Availability

All datasets generated for this study are included in the manuscript and/or the supplementary files.

## Ethics Statement

The study was conducted according to the principles of the Declaration of Helsinki, and it was approved by the Medical Research Council of the Eye, Ear, Nose and Throat Hospital, Fudan University, Shanghai, China (No. KJ2008 01). Informed consent was obtained from each patient before the study's protocol was implemented.

## Author Contributions

LT, L-ML, YX, and H-DD: designed and conceived the experiments. YX, H-DD, DT, DZ, C-YH, and YH: data acquisition. YX, S-JL, JZ, C-WZ, and C-CY: analyzed the experimental data. YX and H-DD: finished the paper. All authors reviewed and validated the manuscript.

### Conflict of Interest Statement

The authors declare that the research was conducted in the absence of any commercial or financial relationships that could be construed as a potential conflict of interest.
